# Mixed reality applications in upper extremity surgery: the future is now

**DOI:** 10.1530/EOR-24-0080

**Published:** 2024-11-08

**Authors:** Daniel B Calem, Przemysław Lubiatowski, Scott Trenhaile, Bruno Gobbato, Ivan Wong, Jawaher Alkhateeb, John Erickson

**Affiliations:** 1 Rutgers New Jersey Medical School, Newark, New Jersey, USA; 2 Sports Trauma and Biomechanics Unit, Rehasport Clinic, Poznań, Poland; 3 Sport Traumatology and Biomechanics Unit, Department of Traumatology, Orthopaedics and Hand Surgery, University of Medical Sciences in Poznań, Poznań, Poland; 4 Orthoillinois, Rockford, Illinois, USA; 5 Departamento de Cirugía Ortopédica, Hospital São José, Jaraguá do Sul, SC, Brasil; 6 Division of Orthopaedics, Department of Surgery, Dalhousie University, Halifax, Nova Scotia, Canada; 7 Overlook Medical Center, Atlantic Health System, Orthopaedic Surgery, New Providence, New Jersey, USA

**Keywords:** augmented reality, mixed reality, preoperative planning, technology, upper extremity surgery

## Abstract

- Mixed reality refers to the integration of virtual reality into the real-world environment. This digital content can be interacted with in real time.
- The emergence of mixed reality technology has been made possible by the introduction of head-mounted displays, which are being utilized across multiple surgical specialties. In upper extremity surgery, mixed reality has widespread applications in trauma, corrective surgery, arthroplasty, arthroscopy, and oncology.
- Preoperatively, mixed reality allows for complex 3D planning.
- Intraoperatively, surgeons can access this 3D data in a sterile environment. While in its infant stages, mixed reality is likely to become a powerful tool for intraoperative guidance and navigation.
- Mixed reality can change the paradigm of communication, as it allows the sharing of visual data from the surgeon’s perspective, enabling remote assistance and participation.

## Introduction

Surgical innovation aims to create value by improving patient outcomes and safety, enhancing surgical techniques, maximizing surgeon efficiency, and minimizing costs. Mixed reality (MR) has emerged as a groundbreaking technology with widespread applications across multiple medical specialties. In 1994, Paul Milgram first described the concept of MR as existing between reality and virtual reality (VR) in a continuum (1). VR refers to complete immersion in a digital world. In augmented reality (AR), virtual objects are superimposed on the real world and anchored in space. In MR, one can interact with these virtual objects in real time.

The emergence of MR has been made possible in recent years through the development of head-mounted displays, such as the Microsoft HoloLens, which was introduced in 2017. These devices provide hands-free holographic information within the user’s visual field and have various sensors that allow the user to interact with virtual objects through voice commands, gaze, and hand gestures. Recently, academic interest in head-mounted displays has increased dramatically (2). This is reflected economically, as the global AR/VR market is projected to grow 350% from $2.7 billion to nearly $10 billion in the next 5 years (3).

These new technologies continue to be adopted across surgical specialties, namely neurosurgery, general surgery, and urology (2, 4, 5, 6). There is also a growing number of applications within orthopedic surgery (7, 8, 9). While the physical world is 3D, most data used by orthopedic surgeons are 2D, from notes and imaging on pages and screens to intraoperative fluoroscopy. MR superimposes 3D data on a 3D world, thereby closing that gap. Preoperatively, MR allows enhanced 3D interaction with CT reconstructions. Intraoperatively, MR can link a preoperative plan with the patient’s anatomy and can be used for navigation, intraoperative guidance, and visualization (10). This has been shown to increase precision, reduce radiation exposure, and/or decrease operative time in various procedure types such as vertebroplasty (11, 12, 13, 14), superior ramus K-wire placement (15, 16), dynamic hip screw placement (17, 18), resection of bony neoplasms (19, 20), and cup placement in total hip arthroplasty (21, 22). Moreover, MR allows access to patient information while maintaining a sterile environment. It also shares information from the surgeon’s point of view and can be used for intraoperative remote guidance, participation, and surgical assistance (23). Postoperatively, AR can better educate patients and integrate them more actively and effectively into the rehabilitation plan (24). Lastly, MR is emerging as a powerful hybrid tool in orthopedic training, combining haptic, visual, and audio technology to create an immersive training environment (25, 26, 27).

While various studies have focused on the role of MR in shoulder arthroplasty (8, 23, 28), many other potential uses in upper extremity surgery remain untapped. As this technology emerges from its infancy, it has the potential to become a powerful tool in increasing surgeon precision and reducing surgical error. While robotic surgery may increase costs, MR head-mounted displays have the potential to be a more cost-saving and space-efficient alternative. This review will focus on a variety of MR applications in upper extremity surgery.

## Trauma

While orthopedic trauma surgery is meticulously tailored to each injury, MR offers intuitive feedback, enhancing the precision and efficiency of orthopedic implant placement. This has extensive applications across orthopedic injuries. Previous research has demonstrated the utility of MR in decreasing operative time, reducing radiation exposure, and improving accuracy in superior ramus K-wire placement and dynamic hip screw placement (15, 16, 17, 18). These benefits can also be utilized in upper extremity trauma. 3D mapping via CT imaging can guide diagnosis, treatment plan selection, and surgical fixation design in fractures of the scapula (29, 30, 31), distal humerus (32), proximal radius, and scaphoid (33, 34). MR allows for the visualization of this 3D imaging in 3D virtual space and has powerful applications in preoperative visualization and planning as well as intraoperative access and guidance. The capacity to better inform the surgeon regarding complicated fracture patterns and deliver an accurate execution of a preoperative plan is perhaps the most useful area of MR in upper extremity surgery.

**Figure 1:**
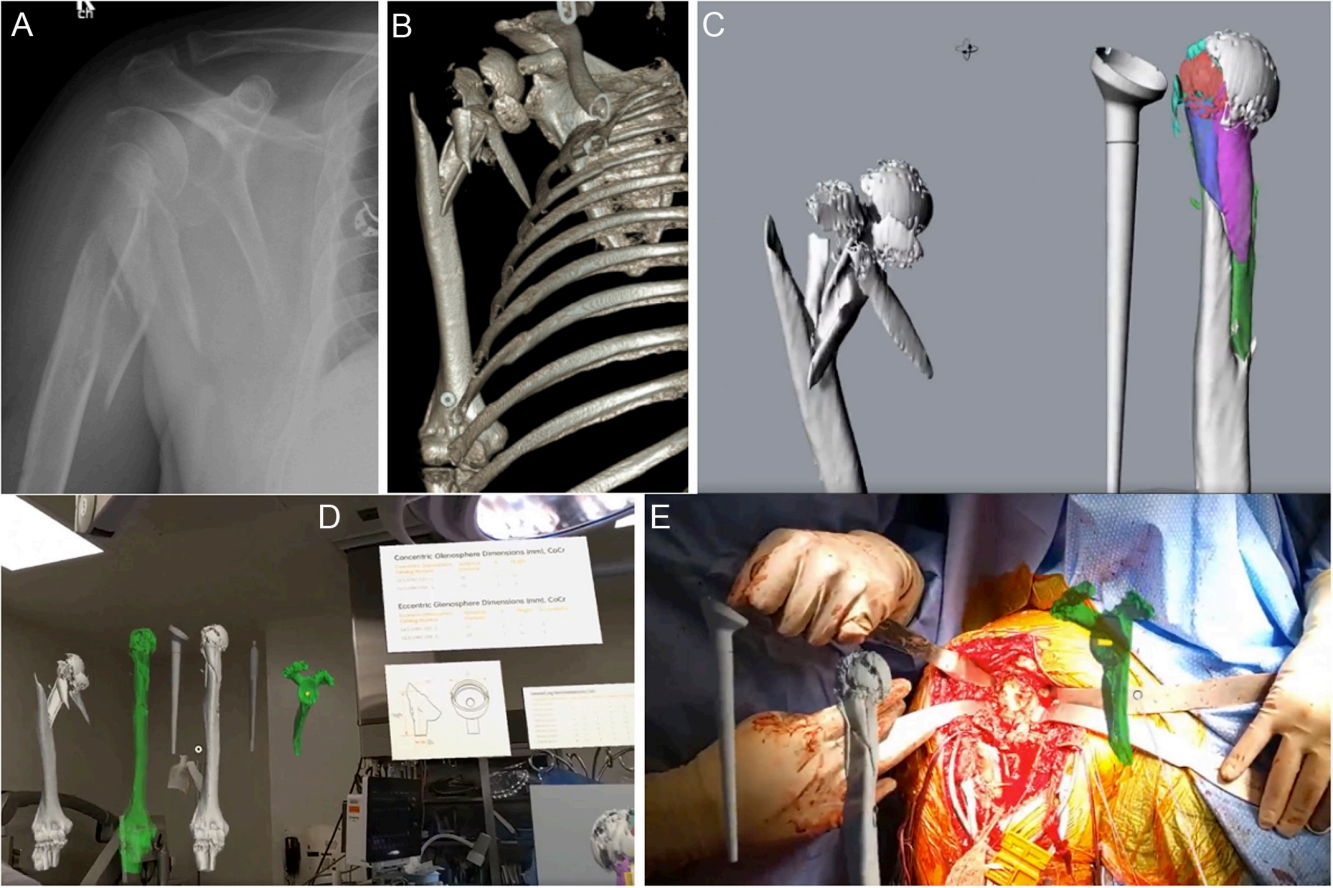
Proximal humerus fracture treated with reverse shoulder arthroplasty using mixed reality. (A) Initial injury anteroposterior (AP) radiograph, (B) 3D CT reconstruction of fracture,and (C) 3D planning to preoperatively plan fracture reduction and stem height. (D) Intraoperative holograms in the operating room, dragged and positioned in space. (E) Positioned holograms to assist in glenoid pin placement, stem placement, and fracture reduction.

**Figure 2:**
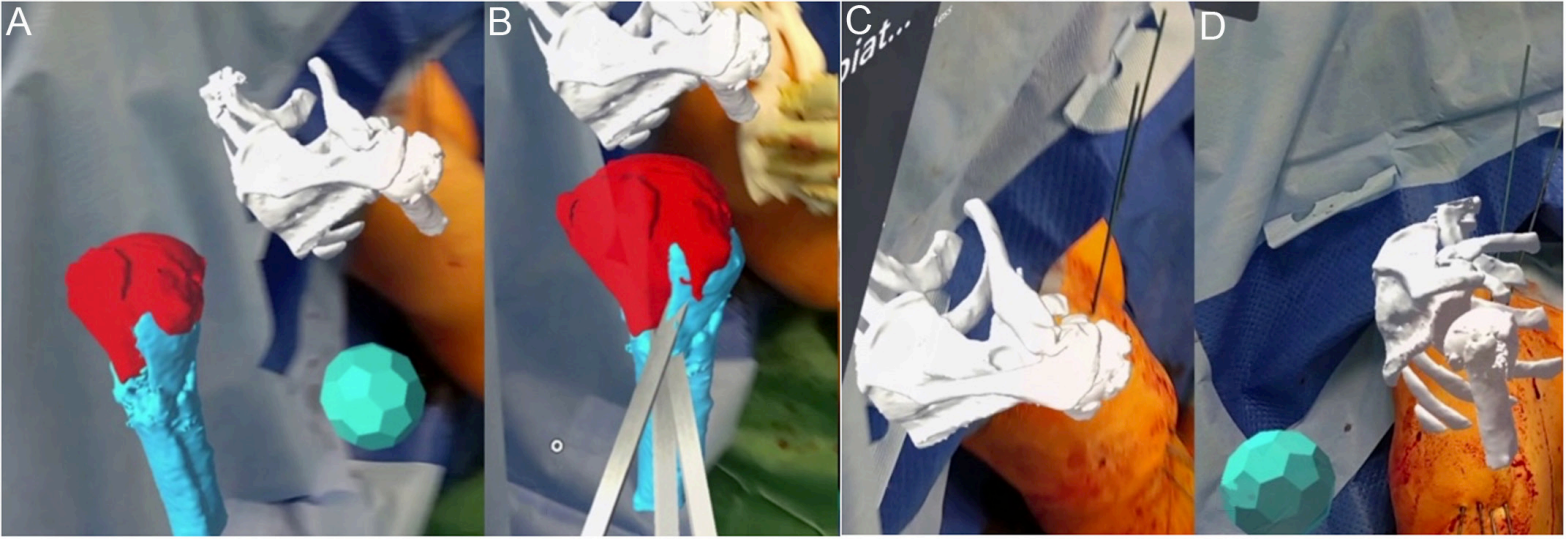
Proximal humerus fracture treated with open reduction internal fixation using mixed reality. (A and B) Intraoperative holograms for use in preoperative planning and closed reduction of a proximal humerus fracture. (C and D) MR can guide percutaneous pin trajectory.

In the case of a complex fracture of the proximal humerus, MR allows for not only preoperative visualization of the fracture pattern but also virtual manipulation of fracture fragments, which can help plan the reduction technique (Fig. 1). 3D implant models can be made digitally with a high level of accuracy, and MR can be utilized to plan in a way that most current planning software does not otherwise allow. These models can be viewed virtually and manipulated to plan for appropriate implant size and positioning (Fig. 1C). Intraoperatively, interactive holograms can be manually dragged and positioned to provide real-time surgical guidance. For example, MR can be used to set the height and version of the humeral stem and assess tuberosity reduction (Fig. 1E). MR can be equally useful in percutaneous fixation of proximal humerus fractures. Holograms allow for a better understanding of fracture lines and displacement, and allow for the proper trajectory of K-wire fixation (Fig. 2).

**Figure 3:**
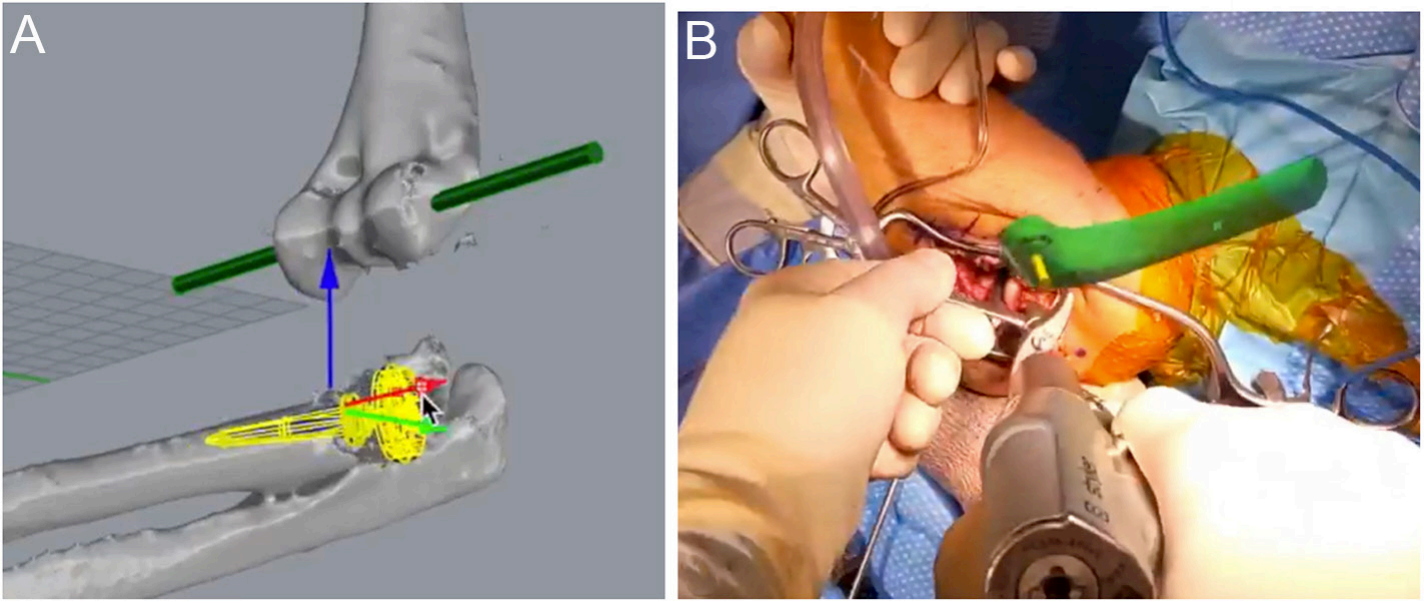
Internal joint stabilizer (IJS) placement to treat elbow instability using mixed reality. (A) 3D preoperative planning of radial head prosthesis size and positioning. (B) Intraoperative MR-assisted guidance of transepicondylar axis pin.

Managing unstable fracture dislocations about the elbow can be challenging. Recently, the internal joint stabilizer (IJS; Skeletal Dynamics), which allows early postoperative motion, has been introduced (35). This device relies on the transepicondylar axis of the distal humerus to obtain an early stable concentric ulnohumeral range of motion. The axis pin placement can be a challenging part of the case but is critical for the outcome. Further surgical treatment of the unstable elbow may rely on the transepicondylar axis for the placement of screws, suture anchors, or tunnels for ligament reconstruction. Intraoperatively, the use of MR as a hologram in the operating field may improve 3D-spatial awareness and increase accuracy and efficiency in drilling the transepicondylar axis. The hologram can be placed immediately adjacent to the distal humerus in the surgical field, displaying the correct trajectory (Fig. 3B). Bone stock is sometimes a limited resource, and obtaining an accurate start point without multiple attempts may be critical. MR can provide preoperative planning and intraoperative assistance for challenging wire placement, proving invaluable in case efficiency and bone preservation. Additionally, MR can be used to preoperatively determine radial head prosthesis size and positioning for radial head arthroplasty (Fig. 3A).

## Osteotomy

One of the most common deformities in the upper extremity is cubitus varus, most commonly as a result of a pediatric supracondylar humerus fracture malunion. Although primarily a cosmetic issue, some patients may experience long-term sequelae, including pain, tardy ulnar nerve palsy, and elbow instability. In cases where the deformity is clinically significant, a closing wedge humeral osteotomy proves to be an effective treatment option. Regardless of the osteotomy method, the rates of complication with this procedure are notably high (36), and postoperative loss of correction is observed in 80% of patients (37). Preoperative planning and accurate correction guidance are therefore crucial.

Extensive research has been conducted on lower limb deformity and osteotomy. Computer-assisted surgery (31) and patient-specific instrumentation (PSI) (38, 39) have both been shown to increase accuracy in proximal tibial osteotomy. However, these methods require costly technology and preoperative preparation. Once the instrument is printed, the angle of the template cannot be adjusted during surgery if necessary. Both methods also necessitate the use of a CT scan, posing a radiation risk to the pediatric population. MR offers a user-friendly alternative by employing virtual wedges that can be created ad hoc at any angle. These wedges can be generated preoperatively for planning or intraoperatively for guidance. The holographic wedge can be accurately placed within the bone at the center of rotation of angulation (CORA) without risk of collision with tissues or other instruments and without enlarging surgical exposure, thus lowering the infection risk. Multiple wedges can be created during surgery, and their sizes can be modified through intuitive gestures of ‘stretching’ and ‘shrinking.’

**Figure 4:**
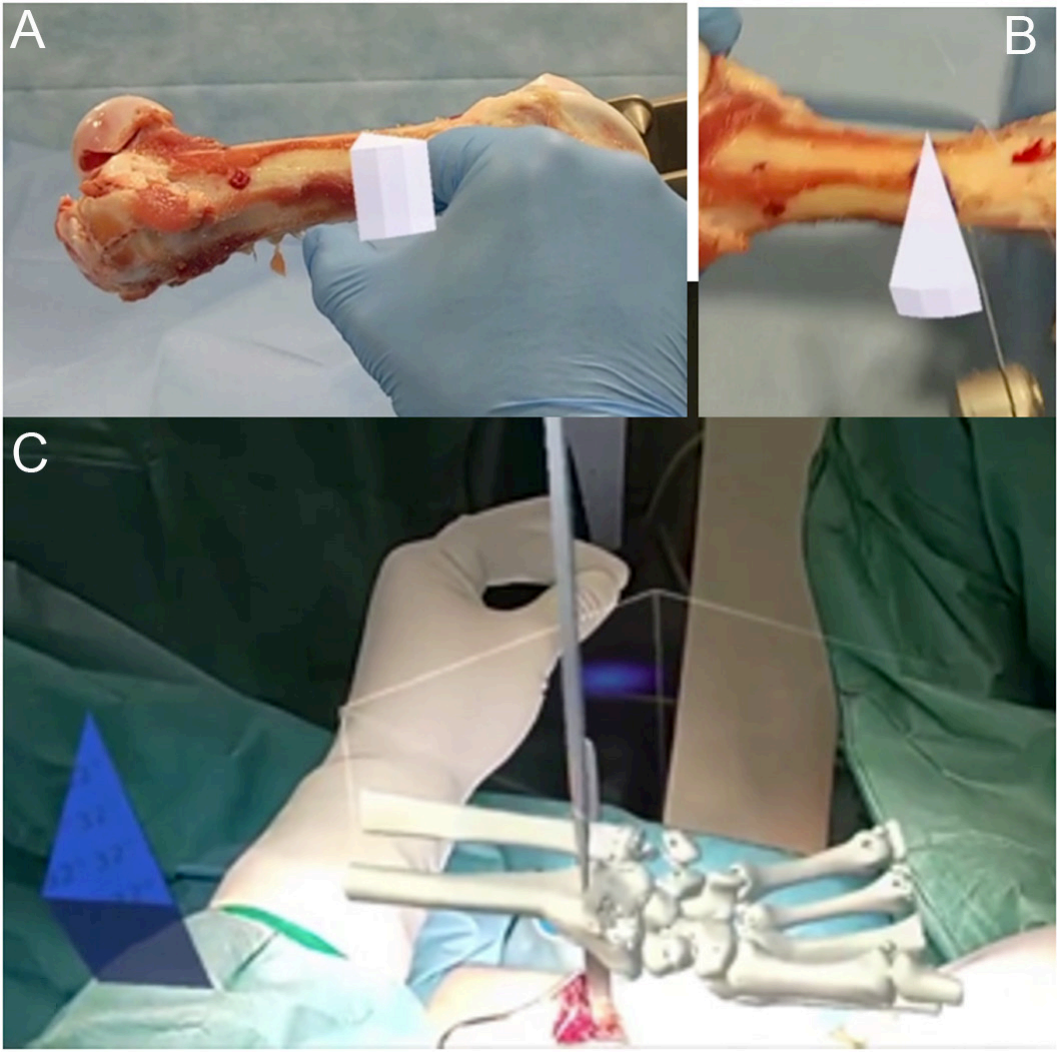
Proximal humerus fracture treated with open reduction internal fixation using mixed reality. (A and B) Intraoperative holograms for use in preoperative planning and closed reduction of a proximal humerus fracture. (C and D) MR can guide percutaneous pin trajectory.

The holographic wedge is not merely a flat template. Due to its polyhedral shape, the lateral surface can aid as a gauge for the oscillating saw (Fig. 4A and B). Our previous research has demonstrated that MR guidance provides superior accuracy and precision compared to visual control and printed templates (40). Results show correction in both the AP and lateral planes. Such instrumentation can be applied in numerous clinical scenarios regardless of the bone or joint in question, including derotational correction and shaping bone graft for open distal radius osteotomy (Fig. 4C).

Another major advantage of MR in osteotomy is during 3D planning. Some deformities require correction in multiple planes. Finding the proper axis of correction of angulation and CORA sometimes involves challenging calculations. These can be particularly difficult in cases of rotational malalignment. MR simplifies these processes by immersing the user in a truly 3D space, rather than projecting 3D objects on a 2D screen. Bilateral comparisons or even mirror images with color-coded models can expedite the planning process. Holographic measurement instruments such as calipers, protractors, and angular wedges can be employed for calculations. All planning can be completed beforehand and easily reproduced in the operating theater.

## Arthroplasty

Shoulder arthroplasty has its challenges, particularly with glenohumeral deformity and, more specifically, glenoid bone loss when preparing for reconstruction of degenerative shoulder arthritis and cuff tear arthropathy. Glenoid deformities are not always fully appreciated on 2D CT scans, which has led to the use of 3D reconstructions to better evaluate glenoid vault shape, depth, and containment of the glenoid bone loss (41).

Accuracy is critical for some of the most challenging glenoids with severe bone loss, and access to bony fixation is crucial for successful surgery. It has been shown that the surgical technique impacts the survivorship of glenoid implants. Surgically addressing an excessively retroverted glenoid by excessive corrective reaming or simply leaving the glenoid component in retroversion can lead to glenoid component migration, loosening, and implant failure (42, 43). Therefore, the goals of glenoid reconstruction include the following: maximizing backside contact of the implant, minimizing bone reaming, restoring version and inclination, preventing cage/peg perforation, maximizing fixation, and preventing bony impingement of implants.

Preoperative planning software has been developed to achieve these goals by using CT scan images to better manage the version, inclination, and overhang of implants intraoperatively. 3D preoperative planning software and the use of patient-specific bone models and transfer devices improved the positioning accuracy of pin placement to guide the placement of glenoid components over the use of handheld instruments alone (44). Several cadaveric studies have demonstrated that AR is highly effective in achieving precise glenoid pin entry points and trajectories (45, 46), as well as accurate overall glenoid component placement (47).

**Figure 5:**
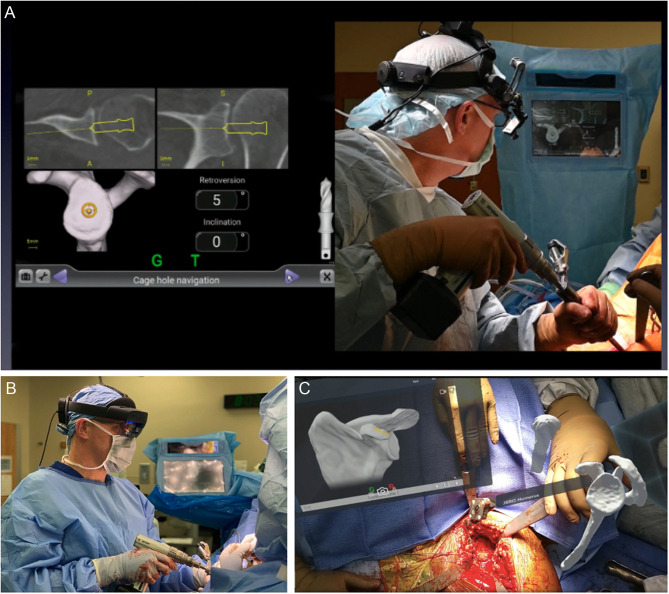
(A) Using traditional navigation techniques, the surgeon must look away from the surgical field while placing the central pin. (B) Head-mounted displays allow the surgeon to look directly into the surgical incision while simultaneously viewing the navigation software, a 3D model of the glenoid humerus, and fluoroscopy within MR. (C) These MR features are shown from the surgeon’s point of view.

Further developments have led to the concept of ‘live’ navigation surgery, which is performed intraoperatively. These systems utilize a 3D CT scan that is segmented for use in the preoperative planning of implants. Intraoperatively, the scan and preoperative plan provide step-by-step guidance throughout the case, including a real-time view of reaming, drilling, screw placement, and implant rotation. One caveat with navigation is that it requires the surgeon to physically look away from the operative field at a sterile electronic tablet to follow the live information that navigation provides (Fig. 5A). MR allows the surgeon to set up their own field of view as they see fit for each individual case (Fig. 5B and C). The view can include a 3D model of the glenoid or humerus, MRI or CT scan images, preoperative planning software, and virtual touch screens of the navigation system. Not only does MR allow access to more information in the sterile field, but it also eliminates the need to create and sterilize 3D-printed models. The surgeon’s focus stays within the surgical wound with improved ergonomics resulting in enhanced visual and tactile cues during the procedure (Fig. 5B).

## Arthroscopy

Arthroscopy relies on optimal portal placement for both instrumentation and visualization. MR can aid in a variety of shoulder and elbow arthroscopic procedures, providing real-time access to preoperative measurements in the sterile field and continuous monitoring of the spatial orientation of arthroscopic instrumentation. This can help obtain ideal portal trajectories relative to patient anatomy, as well as improve surgeon ergonomics and access to visual information and various holographic tools. In our experience, MR has been used in arthroscopic rotator cuff repair, anatomic arthroscopic glenoid reconstruction (AAGR), acromioclavicular (AC) injury, and arthroscopic elbow release to aid in surgeon accuracy and efficiency.

**Figure 6:**
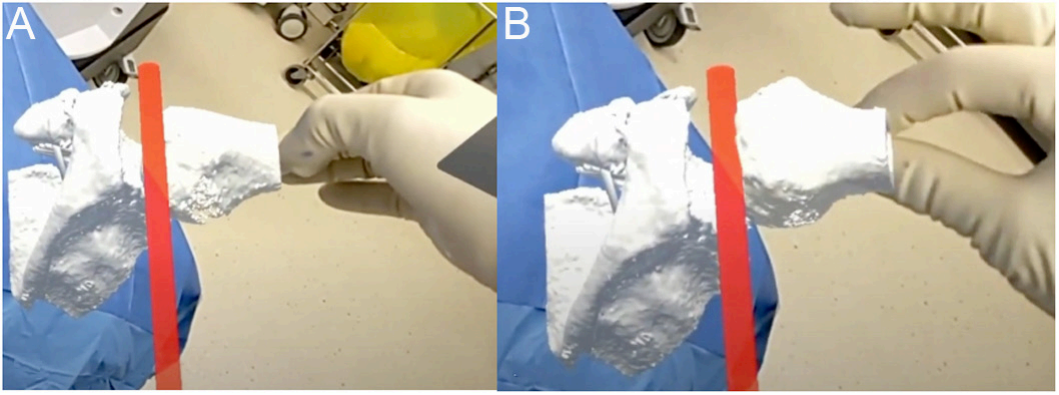
MR in arthroscopic rotator cuff surgery. (A) The patch, represented by the red line, is inserted through the posterior portal. (B) The virtual humerus is rotated to identify the optimal arm position to obtain the maximal greater tuberosity footprint.

MR can be employed in arthroscopic rotator cuff surgery, particularly in cases of massive cuff repair requiring augmentation with a patch. Accessing the subacromial space may not be equally straightforward in all shoulders, despite the consistent placement of portals relative to surface landmarks (48). MR can be used to plan optimal portal placement in both the preoperative and intraoperative settings. A virtual scapula and proximal humerus model can be manipulated to identify the optimal arm position to obtain the maximum footprint for greater tuberosity coverage. It can also help to visualize the optimal trajectories for the viewing and anchor placement portals (Fig. 6).

Managing glenoid bone loss in recurrent shoulder instability presents a significant hurdle for the treating surgeon (49). Arthroscopic techniques to reconstruct the glenoid have gained popularity in recent years after yielding excellent clinical outcomes (50). AAGR is a surgical technique that uses distal tibia allograft to reconstruct the glenoid, thereby allowing preservation of the patient’s native coracoid and subscapularis tendon. A successful technique requires meticulous surgical planning and execution, namely bone loss estimation, arthroscopic portal placement, graft preparation, insertion, positioning, and fixation.

**Figure 7:**
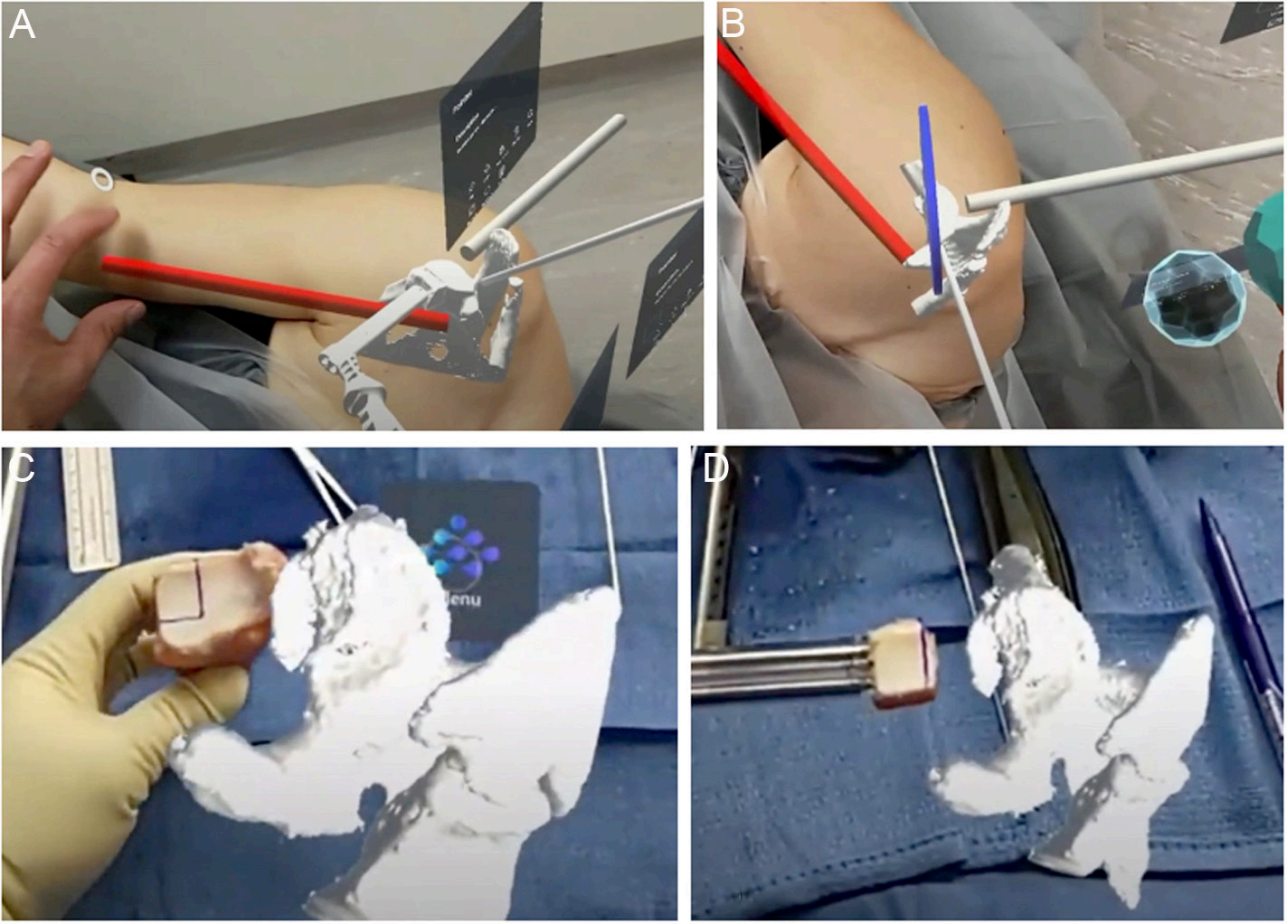
MR use in arthroscopic glenoid reconstruction using distal tibia allograft. (A) The arm can be manipulated to obtain an optimal medial portal position, which is visualized relative to the conjoint tendon, displayed in red. (B) A virtual divider tool can aid in the visualization of the amount of bone to be resected to outline a precise 90-degree bone cut. (C) A holographic model of the glenoid fossa is displayed next to the real-time distal tibia allograft to match the marked bone graft shaping. (D) The allograft is cut and positioned on its inserter. Final adjustments can be made when comparing it to the native glenoid.

Precise portal placement is crucial for the procedure’s success. The medial portal must be placed parallel to the glenoid surface, avoiding the conjoint tendon. The patient’s virtual scapula (containing the glenoid bone loss) can be positioned in space overlying the patient’s shoulder to simulate the arm’s operative position. MR can be used to holographically draw the patient’s conjoint tendon, and the virtual scapula can be manipulated from neutral to adduction to determine the optimal position to displace the conjoint tendon medially and aid in portal placement (Fig. 7A). Furthermore, the glenoid bone loss needs to be refreshed and cut at a perpendicular angle to accommodate the distal tibia allograft. A virtual divider tool can aid in the visualization of the amount of bone to be resected. Additionally, the distal tibia allograft must be contoured to align with the glenoid bone defect, ensuring anatomic restoration of the articular surface (Fig. 7C). This alignment can be assessed intraoperatively using a hologram display of the glenoid fossa model. The bone graft is placed over the defect, and any irregularities in the graft are matched to the glenoid surface precisely.

**Figure 8:**
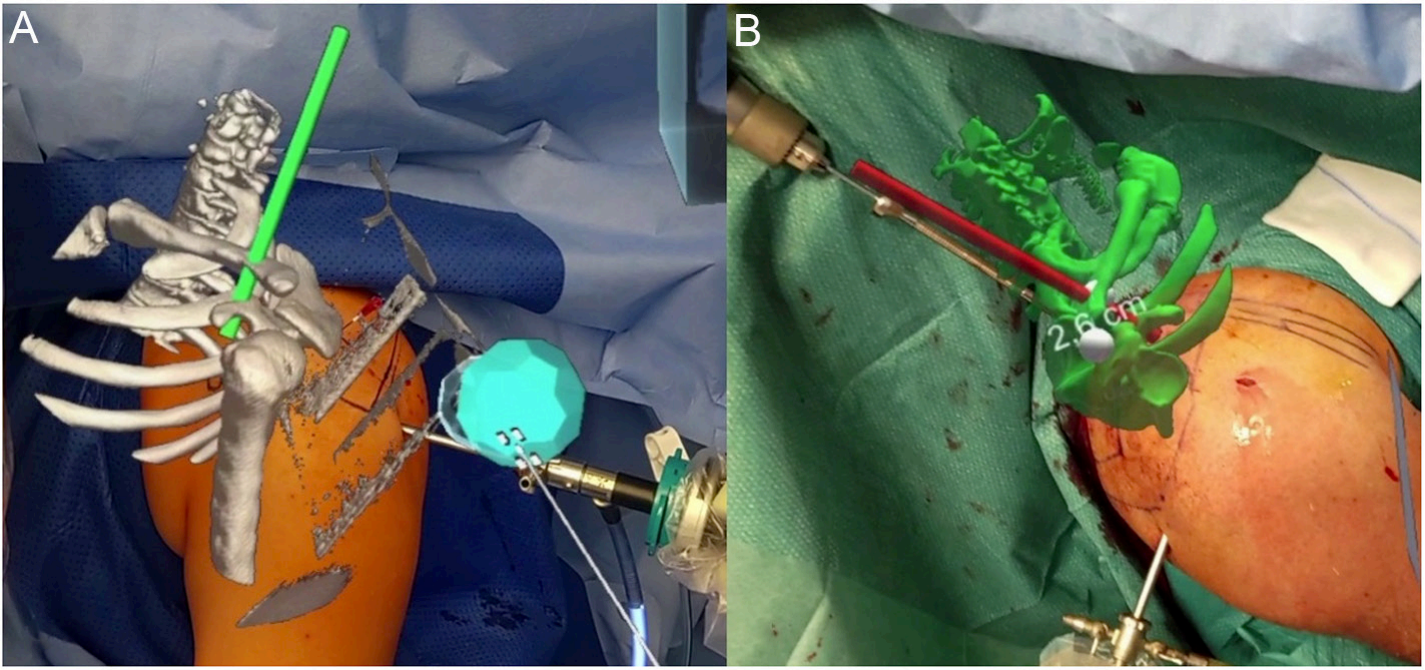
(A and B) MR holograms guiding proper arthroscopic drilling trajectory of the coracoclavicular tunnel.

**Figure 9:**
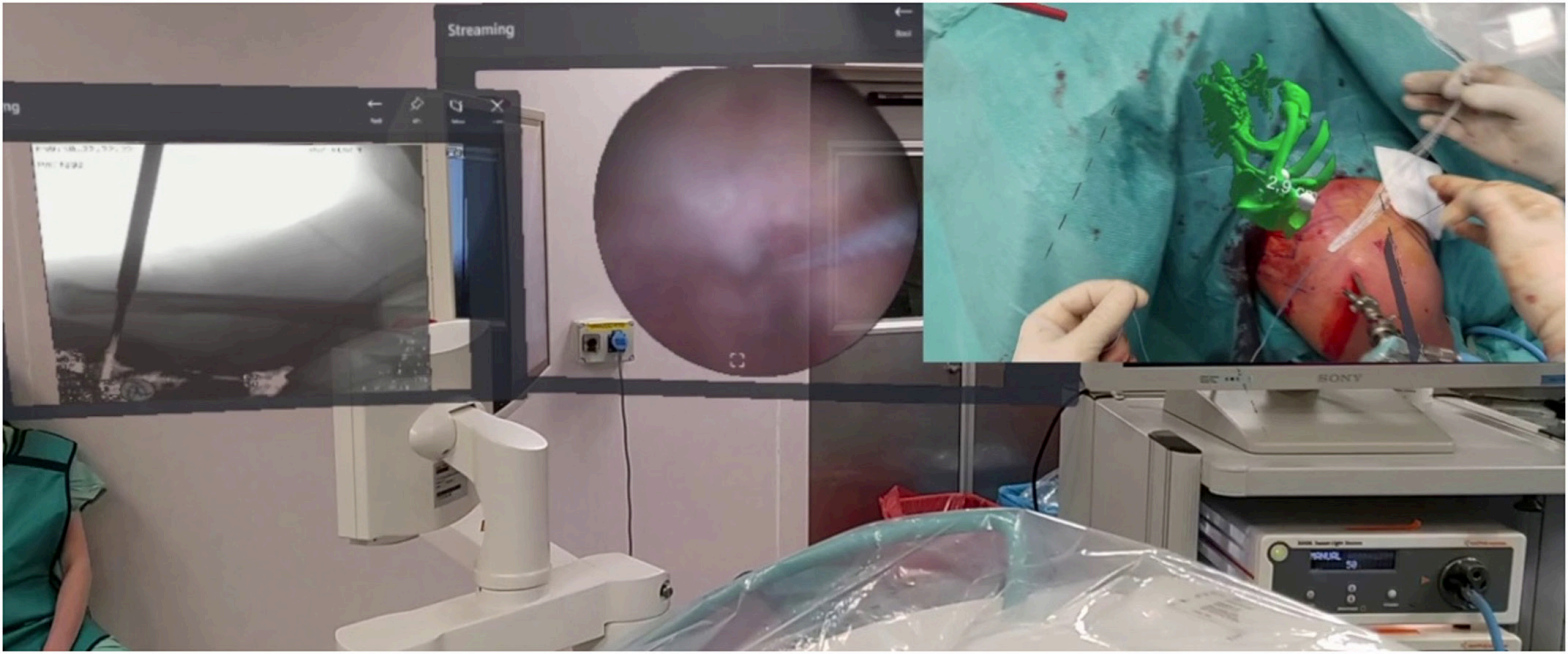
Virtual screens can be placed in any space desired by the surgeon and adjusted in size and distance, thereby providing immediate and convenient access to visual information during arthroscopic surgery and reducing the burden of surgical equipment overload.

MR can also be useful in the arthroscopic management of AC injury. It has been reported that accurate drilling through the coracoid occurs in only 62–84% of cases, leading to a 20–38% re-drilling rate despite the use of guiding instruments (51, 52). While navigation has been shown to be helpful, it is not widely available. In such instances, MR imaging may aid in several ways. It is not yet a substitute for navigation and cannot replace standard guided instrumentation. However, it may significantly improve anatomical spatial awareness. Patient models can be accurately placed over the patient's body with the help of bone rendering. Virtual guides can then be placed to mimic the accurate direction of drilling through the clavicle and coracoid (Fig. 8). Holographic instrumentation can also facilitate measurements of the appropriate distance and location of the clavicle. Furthermore, MR can project a holographic virtual screen. AC joint surgery requires a lot of technical equipment, including an arthroscopic tower and C-arm. Visual access to control both the image intensifier and arthroscopic screen may be cumbersome. Virtual screens for both can be manually placed by the surgeon in the most convenient space in the operating field and adjusted to the size and distance from the eyes (Fig. 9), thereby maximizing surgeon efficiency.

Arthroscopic elbow release is a technically demanding procedure. A major challenge is the safe entry into the joint among numerous vital neurovascular structures. Additionally, one may have to debride outside the joint when performing capsular release and osteophyte or heterotopic ossification (HO) resection, putting these neurovascular structures at risk. 3D planning has historically been projected on 2D screens in the operating room. MR offers the major advantage of projecting 3D holographic models in 3D space overlying the patient’s anatomy.

**Figure 10:**
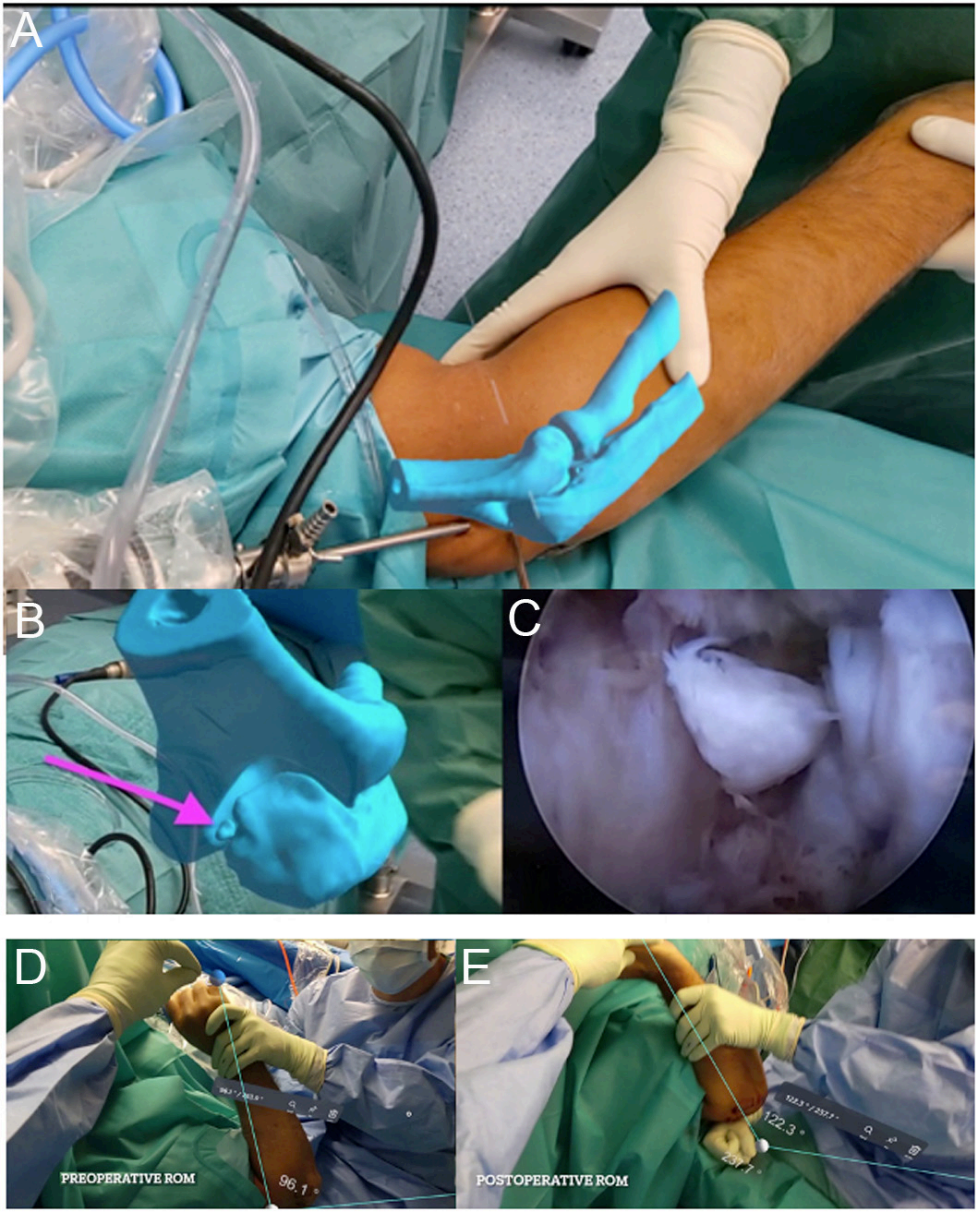
Arthroscopic elbow release using MR. (A) Elbow hologram overlayed over the patient’s anatomy intraoperatively to aid in elbow arthroscopic portal placement. (B and C) This can aid in the removal of hidden loose bodies. MR protractor tool to quantify passive elbow range of motion (D) preoperatively and (E) postoperatively.

Establishing the initial portals and viewing field during elbow arthroscopy requires a “nearly blind” entry into the joint. This requires a high level of hand-eye coordination, spatial orientation, and surgical skill. While training with either sawbones (53) or 3D-printed models (54) can enhance performance for novel elbow arthroscopists, MR offers particular advantages and can be utilized in real-time intraoperatively. Through MR, holograms can be overlaid onto the patient’s anatomy, offering anatomical guidance to aid in a better understanding of portal locations and approaches. Previous research has demonstrated that 3D models and image-enhanced navigation can improve precision during elbow arthroscopy (55, 56), namely with osteophyte and HO removal. MR adds value by allowing constant monitoring of the position and location of the deformity to be removed. Unlike traditional computer screens and printed models, MR allows the surgeon to manipulate the holographic model’s position in space with hand gestures without turning his or her head toward the back screen. The holographic model can be placed either inside the patient’s elbow or just in front of the arthroscopic screen (Fig. 10A). This capability proves particularly beneficial in identifying hidden loose bodies (Fig. 10B). MR also provides other tools as well, such as a holographic pointer, chisel, or protractor. The latter may be used for precise range of motion measurement before and after arthrolysis (Fig. 10D and E).

## Oncology

Orthopedic oncology is a specialized branch of orthopedic surgery focused on the diagnosis and treatment of bone and soft tissue tumors, both benign and malignant. It involves a multidisciplinary approach, with collaboration among oncologists, radiologists, pathologists, and other specialists to provide comprehensive care to patients. MR can be extremely valuable in orthopedic oncology due to the complexity of cases and the distortion of the anatomy.

One of the primary challenges in orthopedic oncology is the complexity of tumor resection and reconstruction. Tumors located in critical anatomical areas such as the pelvis or spine pose significant surgical challenges due to their proximity to vital structures. This is also true in the upper extremity, where complicated neurovascular structures are often displaced by certain tumors. Balancing the goals of achieving oncological clearance while preserving function and quality of life for the patient requires careful planning and innovative surgical techniques.

**Figure 11:**
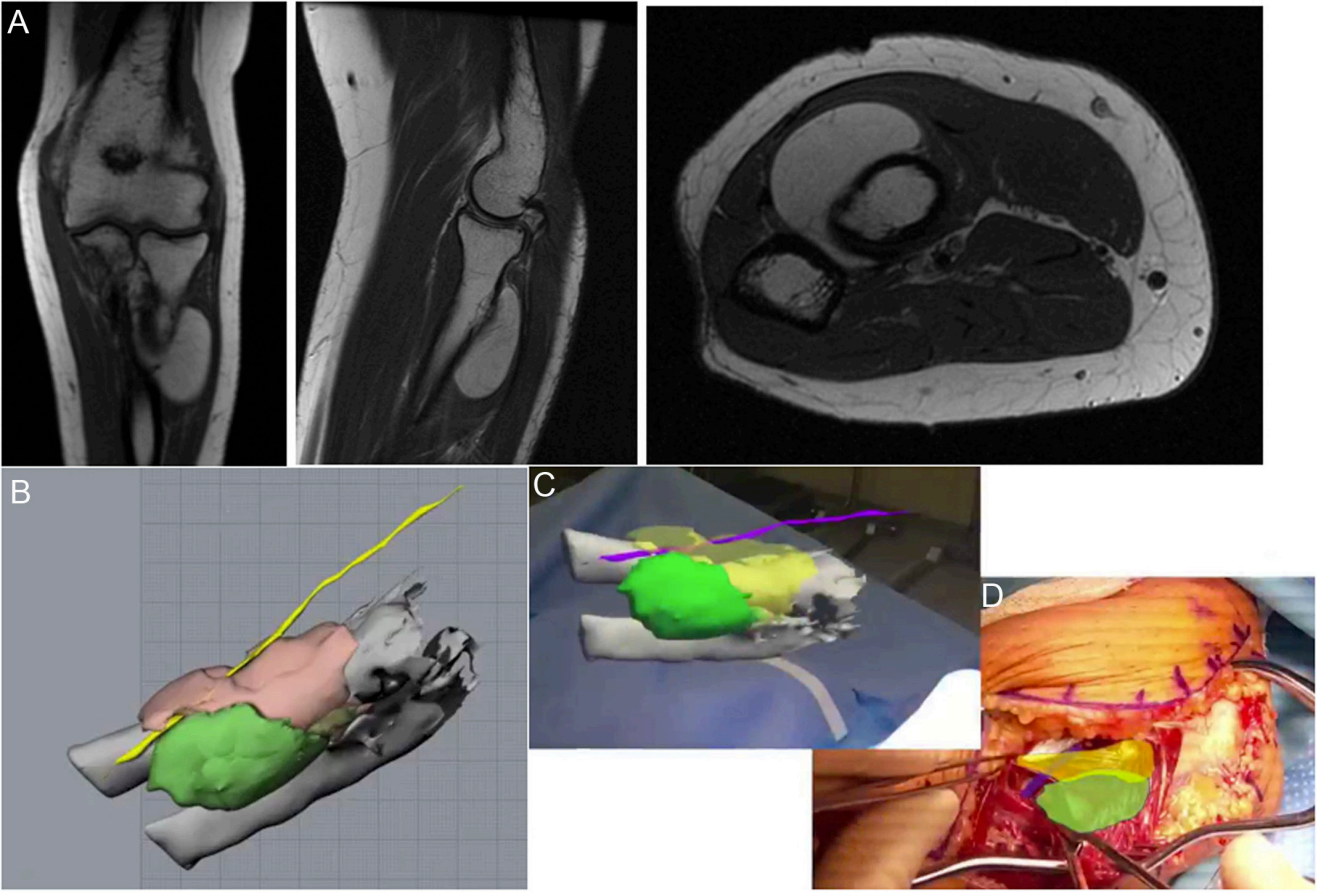
(A) Preoperative MRI demonstrating a well-circumscribed forearm mass within the supinator muscle. (B) MRI was used for 3D preoperative modeling to identify the course of PIN relative to the mass. (C) Intraoperative hologram demonstrating MR assistance. (D) The 3D model is positioned within the surgical field, highlighting the mass (green), supinator (yellow), and PIN (purple).

MR can allow for more thorough 3D planning to assist the surgeon intraoperatively. Both MRI and CT scans can be used to create 3D models of both the tumor and nearby vital structures. For example, Fig. 11 demonstrates the MRI and 3D model of a forearm lipoma within the supinator muscle adjacent to the posterior interosseous nerve (PIN). The 3D model can be used for preoperative planning and moved to be holographically adjacent to the surgical field intraoperatively. The model includes the tumor, supinator muscle, and PIN and can be referenced intraoperatively for safer and more efficient surgical dissection.

### Communication and remote support

Communication is pivotal within surgical teams, and MR introduces a novel dimension to intraoperative communication. By sharing the same visual information through MR headsets, team members can see what the lead surgeon is seeing in both the real-time physical environment and the virtual environment, improving communication and decision-making.

Visual information does not necessarily have to be shared with someone physically in the operating room and instead can be broadcast to surgical assistants participating from a remote location. The lead surgeon’s visual information can be transmitted to co-surgeons, device representatives, or other experts elsewhere, and these consultants can provide their expertise as if they were physically present. Additionally, these consultants can interact not only verbally with the surgeon but can also annotate the surgeon’s visual field with cues or other messages. The visual field of surgical technicians or other assistants wearing HMDs can be interacted with to highlight instruments for the next surgical step, improving operative efficiency. This is particularly beneficial in rural or other geographic areas with limited access to specialized care, democratizing medical expertise. For example, this author used MR from the United States to assist another author in Brazil intraoperatively (57). Gregory *et al.* reported on surgeon experience in an international case series of 13 surgeries using MR headset technology and found that surgeons expressed high satisfaction levels (100%) and resounding intent (94.1%) to integrate MR into future clinical practice (58). Notably, all surgeons found the remote assist functionality and enhanced intraoperative communication to be useful for future practice (58).

The Metaverse, a collective VR shared space, is emerging as a powerful tool in orthopedic surgery. In contrast to MR, the VR space involves complete immersion in a digital world. The Metaverse enables the creation of a fully immersive surgical simulation environment where surgeons can practice and refine their skills without risk to patients. These simulations can mimic a wide range of scenarios, from routine procedures to rare and complex cases, offering a hands-on experience that traditional training methods cannot. In addition, these simulations can be accessed by surgeons in geographically disparate locations, allowing simultaneous training among surgeons across the globe and introducing a novel degree of collaboration not possible in the physical world.

## Limitations

Despite the promising potential of MR in upper extremity surgery, several limitations exist. A recent review compared 3D planning, navigation, PSI, and MR in shoulder arthroplasty and found that each technology comes with unique advantages and disadvantages (59). The implementation of MR comes with increased costs, including the expense of acquiring HMDs, specialized software, and the necessary hardware infrastructure. These expenses can be prohibitive for some healthcare institutions, particularly smaller or resource-limited facilities. Additionally, ongoing maintenance and updates are required to keep the technology functioning optimally, further adding to costs. To date, no formal cost analyses examining MR in orthopedic surgery have been published, and the exact financial implications remain unknown. Future research should address this gap by comparing the costs and benefits of MR against other technological developments such as PSI, computer navigation, and robotics in both upper extremity surgery and orthopedic surgery as a whole.

The complexity of MR systems introduces another layer of challenges. Advances in MR technology necessitate education and training, which can be time-consuming and research-intensive. The learning curve associated with mastering MR tools can initially slow down surgical procedures as teams adapt to new technology. Additionally, incorporating MR into surgical workflows requires added time for surgical planning. Preoperative planning involves creating detailed virtual models, which can be time-consuming. Moreover, the reliance on digital data and technology introduces potential risks, such as technical malfunctions or software glitches. These drawbacks should be weighed against the potential advantages of MR, including more precise surgical execution, precision, and ultimately the potential for improved patient outcomes.

## Conclusion

MR technology is at the forefront of a paradigm shift in orthopedic surgery, offering unprecedented opportunities for enhanced surgical planning and execution. The emergence of head-mounted displays allows the integration of a digital environment into the physical world, providing a platform for more intuitive, precise, and collaborative healthcare delivery. In upper extremity surgery, MR has widespread applications in trauma, arthroplasty, arthroscopy, and corrective surgery, both preoperatively and intraoperatively. While in its nascency, this technology promises to revolutionize the medical field, ultimately leading to better patient care and outcomes.

## ICMJE Conflict of Interest Statement

The authors declare that the study was conducted in the absence of any commercial or financial relationships that could be construed as a potential conflict of interest. ST, IW, and JE and/or their direct family members have some financial interest in a mixed reality company (RSQ Holo). PL is an Associate Editor for EFORT Open Reviews, and was not involved with the peer review of this manuscript.

## Funding Statement

This work did not receive any specific grant from any funding agency in the public, commercial, or not-for-profit sector.
